# Enhanced Tumor Targeting and Antitumor Activity of Methylated β-Cyclodextrin-Threaded Polyrotaxanes by Conjugating Cyclic RGD Peptides

**DOI:** 10.3390/biom14020223

**Published:** 2024-02-15

**Authors:** Shunyao Zhang, Atsushi Tamura, Nobuhiko Yui

**Affiliations:** Department of Organic Biomaterials, Institute of Biomaterials and Bioengineering, Tokyo Medical and Dental University (TMDU), 2-3-10 Kanda-Surugadai, Chiyoda, Tokyo 101-0062, Japan

**Keywords:** polyrotaxane, methylated β-cyclodextrin, cyclic RGD peptide, antitumor activity, tumor targeting

## Abstract

We previously reported that acid-degradable methylated β-cyclodextrins (Me-β-CDs)-threaded polyrotaxanes (Me-PRXs) can induce autophagic cell death through endoplasmic reticulum (ER) stress-related autophagy, even in apoptosis-resistant cells. Hence, Me-PRXs show great potential as anticancer therapeutics. In this study, peptide-supermolecule conjugates were designed to achieve the targeted delivery of Me-PRX to malignant tumors. Arg-Gly-Asp peptides are well-known binding motifs of integrin α_v_β_3_, which is overexpressed on angiogenic sites and many malignant tumors. The tumor-targeted cyclic Arg-Gly-Asp (cRGD) peptide was orthogonally post-modified to Me-PRX via click chemistry. Surface plasmon resonance (SPR) results indicated that cRGD-Me-PRX strongly binds to integrin α_v_β_3_, whereas non-targeted cyclic Arg-Ala-Glu (cRGE) peptide conjugated to Me-PRX (cRGE-Me-PRX) failed to interact with integrins α_v_β_3_. In vitro, cRGD-Me-PRX demonstrated enhanced cellular internalization and antitumor activity in 4T1 cells than that of unmodified Me-PRX and non-targeted cRGE-Me-PRX, due to its ability to recognize integrin α_v_β_3_. Furthermore, cRGD-Me-PRX accumulated effectively in tumors, leading to antitumor effects, and exhibited excellent biocompatibility and safety in vivo. Therefore, cRGD conjugation to enhance selectivity for integrin α_v_β_3_-positive cancer cells is a promising design strategy for Me-PRXs in antitumor therapy.

## 1. Introduction

Cancer is a leading cause of death worldwide, with approximately 19.3 million cases of cancer and approximately 10 million deaths caused by cancer reported in 2020 [1,2]. Traditional cancer treatments cannot meet the demands of cancer therapy because of the increasing number of cancer patients and the intrinsic heterogeneity of malignant tumors [3]. Chemotherapy is widely used in clinical practice as a universally applicable anticancer treatment because it can systemically treat a wide range of cancer types. Despite the remarkable achievements in cancer treatment in recent decades, resistance to classical chemotherapeutic agents remains a major problem [4]. Drug resistance and the resulting ineffectiveness of chemotherapy are the leading causes of cancer relapse and death [5,6,7]. Most chemotherapeutic drugs kill cancer cells by directly or indirectly damaging the DNA of cancer cells through the induction of apoptosis [8,9,10]. Nevertheless, the apoptotic machinery is often mutated in malignant tumors, and the versatility of cancers in evading therapies makes drug resistance more challenging [11]. Therefore, exploiting non-apoptotic programmed cell death to overcome multidrug resistance in cancer cells has received considerable attention [12].

Autophagy is the natural, conserved degradation of cells that removes unnecessary or dysfunctional intracellular components through a lysosome-dependent regulatory mechanism [13]. Increasing evidence has revealed that autophagic dysfunction is associated with various pathological diseases, such as neurodegenerative diseases and cancer [14,15]. Recently, autophagic cell death was identified as a caspase-independent programmed cell death and has received much attention as an effective non-apoptotic pathway of cell death [16,17]. Therefore, the induction of autophagic cell death is considered a potential therapeutic strategy to overcome multidrug resistance in cancer cells, and the development of new drugs that induce autophagic cell death has been widely investigated [18].

Β-Cyclodextrin (β-CD) is a cyclic oligosaccharide comprising seven glucose units connected with α-1,4-linkages. Β-CD and its derivatives can incorporate various low-molecular-weight compounds, such as free cholesterol, into their hydrophobic cavity [19,20]. Recently, the therapeutic applications of β-CD derivatives have received considerable attention [21,22,23], because they improve the pathology of various metabolic diseases, such as Niemann–Pick type C (NPC) disease [24,25,26,27], Alzheimer’s disease [28], and atherosclerosis [29], through the removal of excess free cholesterol. Among various β-CD derivatives, methylated β-CDs (Me-β-CDs) show the highest ability to form inclusion complexes with free cholesterol [30]. Additionally, Me-β-CDs induced apoptosis through interactions with cellular cholesterol and exhibited antitumor effects [31,32]. Interestingly, the modification of Me-β-CDs with tumor-specific targeting molecules such as folate induces autophagic cell death [33,34]. Therefore, tumor-specific ligand-modified Me-β-CDs are potential candidates for new class of antitumor agent that can induce non-apoptotic cell death.

Our group developed acid-degradable polyrotaxanes (PRXs) containing Me-β-CDs (Me-PRXs) as cyclic molecules that can release threaded Me-β-CDs under acidic lysosomal conditions [35,36,37]. Interestingly, the released threaded Me-β-CDs preferentially accumulate in the endoplasmic reticulum (ER) and induced ER stress-mediated autophagic cell death in various cells, such as normal cells and apoptosis-resistant cells [37]. Hence, acid-degradable Me-PRXs are expected to overcome apoptosis resistance and have the potential to treat malignant tumors by triggering autophagic cell death. However, Me-PRXs have low cellular internalization efficiency and lack tumor-targeting ability. Additionally, autophagic cell death induction in normal cells should be avoided. To overcome these issues, our group developed antibody-modified Me-PRX to achieve targeted delivery of Me-PRX [38]. In the case of antibody-modified Me-PRX, the conjugation method is based on the reduction of the thiol group on the antibody, which may lead to insufficient modification with Me-PRX and decrease antibody activity [38].

**Figure 1:**
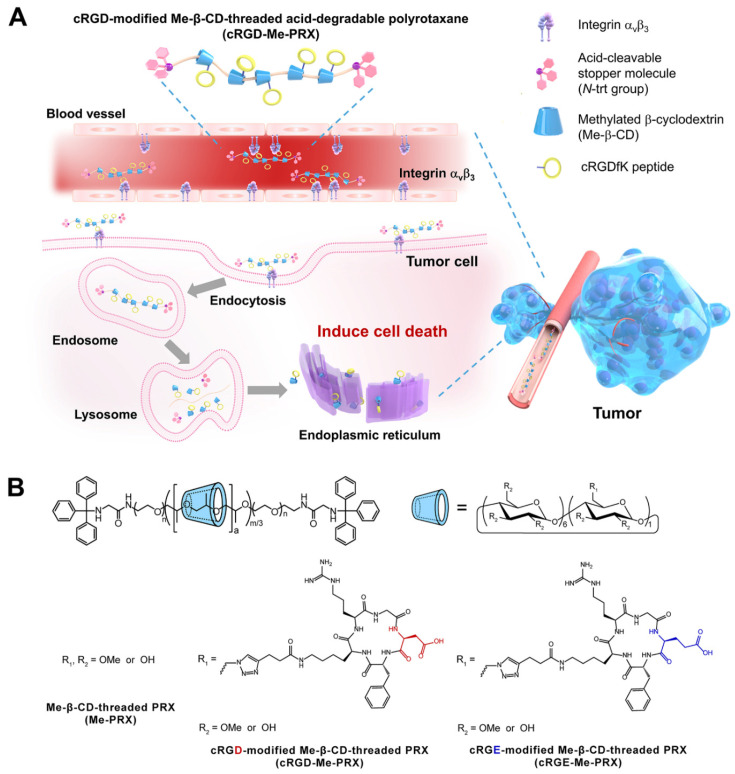
(**A**) Schematic illustration of cRGDfK-conjugated methylated β-CD-threaded polyrotaxane (cRGD-Me-PRX) for specific delivery in integrin α_v_β_3_-overexpressing tumor cells. (**B**) Chemical structures of Me-PRX, cRGD-Me-PRX, and cRGE-Me-PRX.

To overcome these issues, we used tumor-targeting peptides to exploit the anticancer therapeutic potential of Me-PRXs. Targeting peptide-Me-PRX conjugates were designed based on the PRX-containing monoazidated β-CDs (N_3_-PRXs) platform in our previous study [39]. The N_3_-PRXs platform is a highly efficient method for post-modification of targeting ligands and functional molecules on β-CD-based PRXs, because the azide groups on β-CD can easily react with alkynes through click chemistry. Short peptides containing a cyclic Arg-Gly-Asp (RGD) motif (cRGDfK peptide) have selective affinity for integrin α_v_β_3_, which is overexpressed on tumor cells and endothelial cells of tumor neovascularization [40,41]. In this study, the cRGDfK peptide was selected as a tumor-targeting peptide to achieve preferential accumulation of Me-PRX in tumor tissue. cRGDfK peptide-modified Me-PRX (cRGD-Me-PRX) was synthesized for tumor targeting of Me-PRX via the recognition of integrin α_v_β_3_ (Figure 1A). The cellular uptake efficiency and antitumor effect in integrin α_v_β_3_-positive 4T1 tumor-bearing mice were investigated and compared with control Me-PRX modified with the non-targeted peptide cyclic-Arg-Ala-Glu motif (cRGEfK peptide) (Figure 1B). Thus, the specific recognition of cRGD-Me-PRX on tumor cells and the inhibition of tumor growth were verified.

## 2. Materials and Methods

### 2.1. Materials

Acid-degradable PRXs composed of N_3_-β-CD as a cyclic molecule, PEG-*b*-PPG-*b*-PEG (Pluronic P105; BASF, Ludwigshafen, Germany) as an axial polymer, and *N*-Trt groups as acid-labile stopper molecule (N_3_-PRX) were synthesized as previously described [39]. In this study, N_3_-PRX with 14.9 threaded N_3_-β-CDs and a number-average molecular weight of 24,300 was used for the synthesis of cRGD-Me-PRXs. Similarly, acid-degradable PRX with 10.5 threaded β-CDs and a number-average molecular weight of 19,000 was utilized for the synthesis of control Me-PRX. Butynyl cRGDfK and cRGEfK peptides were synthesized and characterized as described in Supporting Information Figures S1 and S2. cRGD-modified Me-β-CD (cRGD-Me-β-CD) was prepared by degrading cRGD-Me-PRX as previously reported procedure [42]. Randomly methylated β-CD (Me-β-CD) and sodium ascorbate were obtained from Merck KGaA (Darmstadt, Germany). Methyl iodide (MeI), copper (II) sulfate pentahydrate, and sodium *N,N*-diethyldithiocarbamate trihydrate (DDC) were obtained from Fujifilm Wako Pure Chemical Industries (Osaka, Japan). All other reagents and solvents were obtained from Fujifilm Wako Pure Chemical and Kanto Chemical (Tokyo, Japan).

### 2.2. Instrumentations

^1^H nuclear magnetic resonance (NMR) spectra were recorded using a Bruker Avance III 400 MHz spectrometer (Bruker BioSpin, Rheinstetten, Germany) in dimethyl sulfoxide (DMSO)-*d*_6_ and D_2_O at 25 °C. The chemical shifts in ^1^H NMR spectra were referenced to DMSO (2.5 ppm in DMSO-*d*_6_) and HDO (4.65 ppm in D_2_O). Size exclusion chromatography (SEC) was performed using a Prominence-i LC-2030 Plus system (Shimadzu, Kyoto, Japan) equipped with an RID-20A refractive index detector and a combination of TSKgel α-4000 and α-2500 columns (300 mm length, 7.8 mm internal diameter; Tosoh, Tokyo, Japan). Sample solutions were injected into the system and eluted with DMSO containing 10 mM LiBr at a flow rate of 0.35 mL/min at 60 °C. The UV–vis absorption of the solutions was measured using a V-550 UV–vis spectrophotometer (Jasco, Tokyo, Japan). Fourier-transform infrared (FT-IR) spectra were recorded using a Spectrum 100 FTIR spectrometer (Perkin Elmer, Wellesley, MA, USA) equipped with a HgCdTe (MCT) detector. The sample powders were ground with KBr to prepare the pellets for analysis.

### 2.3. Synthesis of Methylated PRXs

The synthetic procedure for Me-PRX has been described in previous reports [36,37]. Briefly, the precursor N_3_-PRX (300 mg, 0.14 mmol of threading β-CD) was dissolved in dehydrated DMSO (15 mL). Powdered NaOH (148.8 mg, 7.44 mmol, 2 mol equivalent to hydroxy groups) and MeI (155.3 μL, 2.49 mmol, 0.67 mol equivalent to hydroxy groups) were successively added to the solution. The resulting solution was vigorously stirred for 1 h at room temperature. After the reaction, the solution was diluted with water and dialyzed using Spectra/Por 1 (molecular weight cut-off of 6000–8000; Spectrum Laboratories, Rancho Dominguez, CA, USA) against water for 3 days at 4 °C. The recovered solution was freeze-dried to obtain the Me-N_3_-PRX (280 mg, 85.6% yield). PRX without azide groups was used to synthesize the control Me-PRX in the same manner. The ^1^H NMR spectra of Me-N_3_-PRX and Me-PRX in D_2_O were measured, and the number of methyl groups modified on PRXs was calculated from the ^1^H NMR peak area by subtracting the integral ratio at 3.0–4.0 ppm in the precursor PRXs from the integral ratio at 3.0−4.0 ppm in the Me-PRXs [36]. *M*_n_ was calculated based on the number of threaded β-CDs and methyl groups in the Me-PRXs.

### 2.4. Modification of cRGDfK Peptide

Copper (I)-catalyzed azide–alkyne cycloaddition (CuAAC) was performed as follows: Firstly, N_3_-Me-PRX (100 mg, 3.77 μmol PRX and 54.69 μmol N_3_ group) and butynyl-cRGDfK peptide (65.3 mg, 82.03 μmol) were dissolved in 100 mM phosphate buffer (5 mL). Copper (II) sulfate pentahydrate (13.65 mg, 54.69 μmol) and sodium ascorbate (21.67 mg, 109.37 μmol) were dissolved in cold water (1 mL) added to the solution, which was then stirred for 16 h at room temperature. After completion of the reaction, the mixture was diluted to a final volume of 50 mL. Subsequently, DDC (24.6 mg, 109.4 μmol, 2 mol equivalent of Cu^2+^) was added to the solution and stirred for 30 min to precipitate Cu^2+^ [43]. The yellow supernatant was collected and dialyzed using Spectra/Por 1 against methanol for two days, followed by water for two days. The recovered solution was then freeze-dried to obtain cRGDfK peptide-conjugated Me-PRX (cRGD-Me-PRX; 112 mg, 79.6% yield).

### 2.5. Binding Affinity of cRGD-Me-PRXs with Integrin α_v_β_3_ by Surface Plasmon Resonance (SPR) Measurements

Binding affinity of cRGD-Me-PRXs to recombinant integrin α_v_β_3_ was analyzed by SPR using a Biacore X100 instrument (Cytiva, Marlborough, MA, USA). Recombinant mouse integrin α_v_β_3_ (R&D Systems, Minneapolis, MN, USA) containing N-terminal hexahistidine (His) peptide was immobilized on Sensor Chip NTA (Cytiva) using NTA Reagent Kit containing 0.5 mM NiCl_2_, and 350 mM EDTA (Cytiva). His-tagged integrin α_v_β_3_ was captured on Sensor Chip NTA by the chelation of Ni^2+^ with NTA on the surface and hexahistidine in integrin α_v_β_3_ [44,45]. On the NTA chips, 0.5 mM NiCl_2_ was first injected at 15 μL/min for 1 min. Integrin α_v_β_3_ dissolved in the running buffer (HBS-P; Cytiva) containing 1 mM MnCl_2_ and 1 mM MgCl_2_ at a concentration of 250 ng/mL was then injected at 30 μL/min for 3 min. After that, response unit (RU) of the captured integrin α_v_β_3_ reached approximately 480 RU. cRGD-Me-PRXs dissolved in running buffer were injected at 30 μL/min with an injection time of 2 min and dissociation time of 10 min. The NTA chips were regenerated by injecting 350 mM EDTA solution after each binding assay at 30 μL/min for 1 min. The sensorgrams were analyzed using Biacore X100 evaluation software (version 2.0.1, Cytiva).

### 2.6. Cell Culture

4T1 cells, the murine mammary adenocarcinoma cell line, were obtained from the American Type Culture Collection (ATCC, Manassas, VA, USA). The cells were cultured in Dulbecco’s modified Eagle’s medium (DMEM; Fujifilm Wako Pure Chemical, Komono-cho, Japan) supplemented with 10% heat-inactivated fetal bovine serum (FBS; Gibco, Grand Island, NY, USA), 100 U/mL penicillin (Fujifilm Wako Pure Chemical), and 100 µg/mL streptomycin (Fujifilm Wako Pure Chemical) in 5% CO_2_ at 37 °C.

### 2.7. In Vitro Cytotoxicity

4T1 cells were seeded in a 96-well plate at a density of 1 × 10^4^ cells/well and incubated overnight. cRGD-Me-PRXs were added to each well and incubated for 48 h. After incubation, Cell Counting Kit-8 reagent (Dojindo Laboratories, Kumamoto, Japan) (10 μL) was added to each well, and the cells were incubated for 2 h at 37 °C. The absorbance of each well was measured at 450 nm using a Varioskan LUX multimode microplate reader (Thermo Scientific, Waltham, MA, USA). Cell viability was calculated by comparing the absorbance of the treated cells with that of the untreated cells.

To investigate the induction of autophagic cell death by cRGD-Me-PRXs, 4T1cells were pretreated with 2 mM 3-methyladenine (3-MA; Fujifilm Wako Pure Chemical) for 1 h, followed by treatment with cRGD-Me-PRXs (100 μM threaded β-CD) in the presence of 3-MA for 48 h at 37 °C. Cell viability was determined as described above.

### 2.8. Expression Analysis of Integrin α_V_ and β_3_

4T1 cells were treated with phycoerythrin (PE)-labeled anti-mouse CD51 (integrin α_V_; clone: RMV-7, BioLegend, San Diego, CA, USA), anti-mouse CD61 (integrin β3; clone: 2C9.G2 (HMβ3-1), BioLegend), and isotype control antibodies (clone: RTK2071 and HKT888, BioLegend) on ice for 40 min. The cells were then washed twice with Dulbecco’s PBS (D-PBS; Fujifilm Wako Pure Chemical) and resuspended in D-PBS containing 0.5% bovine serum albumin (BSA) solution. The fluorescence intensity of the cells was measured using a BD FACS Canto II flow cytometer (BD Biosciences, Bedford, MA, USA). A total of 1 × 10^4^ cells were counted for each sample, and the fluorescence intensity of the cell population was determined using the DIVA software (BD Biosciences).

### 2.9. Cellular Association Analysis Using Flow Cytometry

4T1 cells were seeded in 24-well plates at a density of 5 × 10^4^ cells/well and incubated overnight. The cells were then cultured in a medium containing Cy5.5-labeled PRXs for 3 h at 37 °C. Subsequently, the cells were harvested and collected via centrifugation (1500 rpm, 3 min, 4 °C). The cells were then washed with D-PBS containing 0.1% BSA and passed through a 35 μm cell strainer (Corning, Corning, NY, USA). The fluorescence intensity of the cells was measured using a BD FACS Canto II flow cytometer as described above.

### 2.10. Intracellular Distribution Analysis Using Confocal Laser Scanning Microscopy

4T1 cells were seeded in a 35 mm glass-bottomed dish (diameter of glass area: 12 mm; Iwaki, Tokyo, Japan) at a density of 1.0 × 10^4^ cells/dish and incubated overnight. The cells were then cultured in a medium containing Cy5.5-labeled PRXs for 3 h at 37 °C. The cells were stained with LysoBrite Red and ER Red (AAT Bioquest, Sunnyvale, CA, USA), respectively, following the manufacturer’s instructions at 37 °C for 10 min. Cells were stained with 1 μg/mL Hoechst 33342 (Dojindo Laboratories) at 37 °C for 10 min. Confocal laser scanning microscopy (CLSM) was performed using FluoView FV10i (Olympus, Tokyo, Japan). The excitation and emission wavelengths of Hoechst 33342, Cy5.5-labeled PRXs, and LysoBrite Red and ER Red were 352 and 461 nm, 673 and 707 nm, and 575 and 590 nm, respectively. The colocalization of Cy5.5-labeled polymers with LysoBrite Red and ER Red was quantified using Fiji’s Coloc 2 plugin to calculate Pearson’s correlation coefficient [46].

### 2.11. Downregulation of Integrin α_V_ on 4T1 Cells by siRNA

siRNAs against murine integrin subunit alpha V (siItgav; sense: 5′-GACUUAGUUGUAGGAGCUU-3′, antisense: 5′-AAGCUCCUACAACUAAGUC-3′) and Mission siRNA universal negative control were purchased from Merck. The siRNAs were transfected into 4T1 cells using the Lipofectamine 3000 Transfection Reagent (Thermo Fisher Scientific) at 30 nM for 24 h according to the manufacturer’s instructions. Total RNA was extracted from 4T1 cells using the FastGene RNA Premium Kit (Nippon Genetics, Tokyo, Japan) following the manufacturer’s instructions. The total RNA concentration was measured using a NanoDrop One spectrophotometer (Thermo Fisher Scientific). Total RNA was then converted into cDNA using ReverTra Ace qPCR RT master mix (Toyobo, Osaka, Japan). RT-PCR was performed using the Thunderbird SYBR qPCR mix (Toyobo) on a CFX Connect Real-Time System (Bio-Rad, Hercules, CA, USA). The PCR primer sequences were as follows: murine integrin subunit alpha V (*Itgav*), forward: 5′-GCTTAAAGGCAGATGGCAAG-3′, reverse: 5′-AAATGGTGATGGGAGTGAGC-3′, murine glyceraldehyde 3-phosphate dehydrogenase (*Gapdh*) forward: 5′-AACTTTGGCATTGTGGAAGG-3′, reverse: 5′-ACACATTGGGGGTAGGAACA-3′. The PCR cycling conditions included a pre-denaturation at 95 °C for 1 min, followed by 40 cycles of denaturation at 95 °C for 15 s and extension at 60 °C for 30 s. The mRNA expression levels in each sample were determined by the comparative CT (ΔΔCT) method and were normalized to the expression levels of *Gapdh*.

### 2.12. Biodistribution of Me-PRXs

Animal experiments were approved by the Institutional Animal Care and Use Committee of Tokyo Medical and Dental University. Wild-type BALB/c mice (female, 8-weeks-old) were purchased from Japan SLC (Shizuoka, Japan). Cy5.5-labeled PRXs and CDs were intravenously administered to the mice at a dose of 300 mg/kg. After 24 h, the mice were anesthetized with an intraperitoneal injection of an anesthetized solution containing medetomidine hydrochloride (0.75 mg/kg), midazolam (4 mg/kg), and butorphanol tartrate (5 mg/kg). Blood was collected from the inferior vena cava, and plasma was collected after centrifugation (4000 rpm, 15 min). The mice were then perfused with saline, and the hearts, livers, lungs, spleens, kidneys, and tumors were harvested. The collected tissues (approximately 50 mg) were homogenized using a bead beater-type homogenizer (Beads Crusher μT-12; Taitec, Saitama, Japan) at 3000 rpm for 45 s. After centrifuging the homogenates (8000 rpm, 10 min), the supernatant was collected. The fluorescence intensities of the plasma and tissue homogenates were measured using Varioskan LUX at excitation and emission wavelengths of 673 and 707 nm, respectively. The amount of accumulated PRXs in the plasma and each tissue was calculated using the standard curve of Cy5.5-labeled Me-PRXs and Me-β-CDs, and the mean percentage of the injected dose per gram of tissue (ID %/g tissue) was determined for each sample.

### 2.13. Antitumor Activity Assay in 4T1 Tumor-Bearing Mice

The 4T1 cells (1 × 10^6^ cells) dispersed in DMEM (200 μL) were subcutaneously inoculated into the right hind limb abdomen of female BALB/c mice (8 weeks) to form a subcutaneous breast tumor model. After seven days, the tumor-bearing mice were randomly divided into five groups when the tumor volume reached 80–100 mm^3^. Saline, Me-PRX, cRGD-Me-PRX, cRGE-Me-PRX and cRGD-Me-β-CD were intravenously injected into the tail vein of the mice on days 0, 3, and 6 at a dose of 30 mg/kg. The length and width of the tumors were measured every two days using an electronic caliper. The tumor size was calculated as (width)^2^ × length/2. When the tumor volume of the saline group reached 2000 mm^3^, the mice were sacrificed and the tumor tissues were isolated and weighed.

The tumor tissues were dissected from mice, fixed in neutral buffered 10% formalin solution (Fujifilm Wako Pure Chemical) for 1 d, and embedded in paraffin. The collected tumor tissues were cut into prepared tissue sections and stained with hematoxylin and eosin (H&E) to assess histological alterations under a bright-field microscope.

### 2.14. Blood Chemical Analysis

The PRXs dissolved in PBS were intravenously administered at a dose of 500 mg/kg to female BALB/c mice (8 weeks). At 24 h after administration, the mice were sacrificed and blood was collected from the inferior vena cava, combined with EDTA solution, and centrifuged at 1500× *g* for 15 min at 4 °C to collect the plasma. Plasma levels of blood urea nitrogen (BUN), creatinine (CRE), aspartate aminotransferase (AST), alanine aminotransferase (ALT), free cholesterol (F-CHO), cholesteryl esters (E-CHO), triglycerides (TG), and glucose (GLU) were measured using a Hitachi 7180 automatic analyzer (Hitachi, Tokyo, Japan). In addition, the mice were perfused with saline, and the major organs (heart, liver, spleen, lungs, and kidneys) were collected. The organs were fixed in 10% formalin solution for 1 d and embedded in paraffin. The paraffin blocks were cut into 3 μm sections, which were successively stained with H&E solution to assess histological alterations by bright-field microscope.

### 2.15. Statistical Analysis

Data are expressed as the mean ± standard deviation (SD). Statistical analyses were performed using GraphPad Prism 9.5.1 (GraphPad Software, San Diego, CA, USA). To compare two groups, statistical differences were analyzed using a two-tailed unpaired Student’s *t*-test. To compare three or more groups, statistical differences between the means of multiple groups were analyzed using one-way analysis of variance, followed by Tukey’s post hoc multiple comparisons test. Statistical significance was set at *p* < 0.05.

## 3. Results and Discussion

### 3.1. Synthesis and Characterization of cRGD-Me-PRX

A methyl group was first introduced into N_3_-PRX to increase the ability of β-CD to encapsulate cholesterol (Supporting Information). After modification with the methyl group, no peaks corresponding to free β-CD or the axle polymer were observed in the SEC charts of Me-PRX, N_3_-PRX and Me-N_3_-PRX in DMSO (Supporting Information Figure S3), indicating that the methyl group was successfully modified in the PRXs without degradation. To improve the tumor-targeting ability of Me-N_3_-PRX, cRGDfK peptide was applied to Me-N_3_-PRX via CuAAc (Figure 1B), because the RGD sequence has the superior ability of integrin α_v_β_3_ receptor recognition [47]. To compare the tumor-targeting ability of cRGD-Me-PRX, a non-targeting cRGE-Me-PRX was also prepared (Figure 1B). The non-targeting ligand cRGEfK was synthesized by substituting aspartic acid (D) in RGD by glutamic acid (E), which has similar charged functional groups, but may abolish specific binding to integrin α_v_β_3_ [48]. Me-PRXs without peptide modification were prepared as a control (Figure 1B).

**Figure 2:**
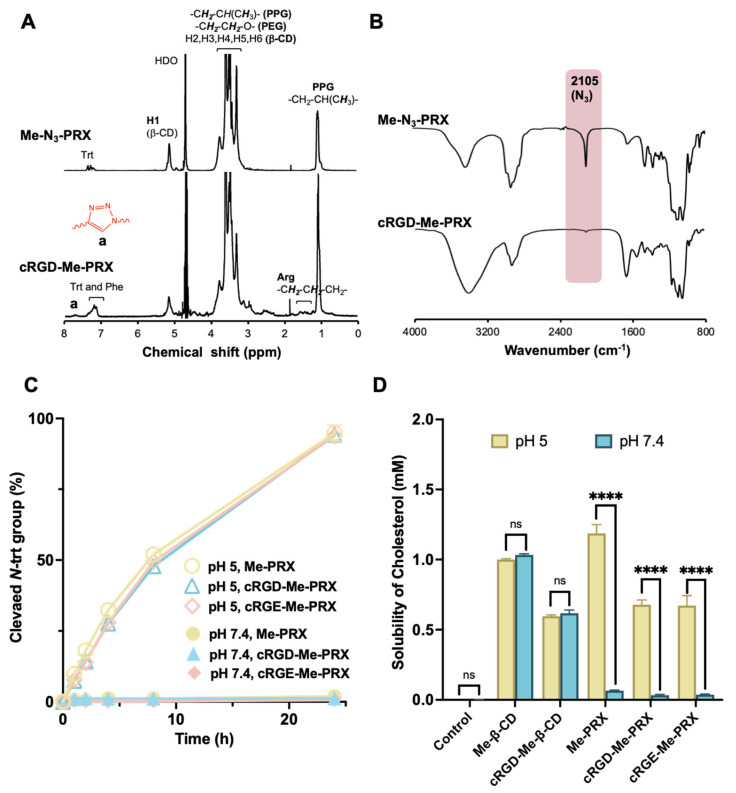
(**A**) ^1^H NMR spectra of Me- N_3_-PRX and cRGD-Me-PRX in D_2_O at 25 °C. (**B**) FT-IR spectra of Me-N_3_-PRX and cRGD-Me-PRX. (**C**) Time course of the cleavage of *N*-Trt end groups in Me-PRX, cRGD-Me-PRX, and cRGE-Me-PRX at pH 7.4 (open symbols) and pH 5.0 (closed symbols) at 37 °C. The data are expressed as the mean ± SD (n = 3). (**D**) Phase-solubility diagrams of cholesterol with Me-β-CD, cRGD-Me-β-CD, Me-PRX, cRGD-Me-PRX, and cRGE-Me-PRX at pH 7.4 (brown bars) and pH 5.0 (blue bars). The solubility of cholesterol was determined after incubation for 24 h at 37 °C. The data are expressed as the mean ± SD (n = 3; **** *p* < 0.0001; ns: not significant).

cRGD-Me-PRX and cRGE-PRX were characterized using ^1^H NMR and FT-IR spectroscopies (Figure 2A,B). In the ^1^H NMR spectrum of cRGD-PRX, a peak corresponding to the triazole rings was observed at 7.72 ppm. Additionally, in the FT-IR spectra, the peak correspond to azide group at 2105 cm^−1^ was disappeared after the modification of cRGDfK. Based on these results, we conclude that cRGD-Me-PRX was successfully synthesized. The number of modified methyl groups and peptide molecules in cRGD-Me-PRX and cRGE-PRX were determined using ^1^H NMR spectroscopy (Figure 2A, Table 1). Similarly, cRGE-Me-PRX and Me-PRX were characterized using ^1^H NMR and FT-IR (Supporting Information Figure S4). As the number of charged peptide molecules and methyl groups on threaded β-CDs may affect their ability to form an inclusion complex with cholesterol, the number of modified peptides and methyl groups in the control PRXs should be the same. Based on the results of ^1^H NMR, cRGE-Me-PRXs with similar ligand numbers and the same number of methyl groups were synthesized (Table 1). Me-PRXs with a small number of methyl groups were synthesized in this study (Table 1). Moreover, cRGD-modified Me-β-CD was prepared by degrading cRGD-Me-PRX (Table 1).

The modification of charged peptides may affect the physicochemical properties of Me-PRXs, such as acid-induced dissociation (release of threaded Me-β-CDs), stability of Me-PRXs under physiological conditions, and the ability of threaded β-CDs to encapsulate cholesterol after release. Acid-induced dissociation of PRX occurs through protonation of the terminal *N*-Trt groups, resulting in the liberation of *N*-triphenylmethanol (Trt-OH) from the terminals of the axial polymer [35,49]. The detailed pH dependency of the acid-induced dissociation of PRXs is described in our previous papers [35,49]. Additionally, the acid-induced dissociation of PRX is not affected by the number of chemical modifications and the type of modified functional groups [35,37,39]. However, the modification of cRGD or cRGE peptide might alter the acid-induced dissociation of PRXs. Therefore, the stability and acid-induced dissociation of cRGD-Me-PRX were evaluated by incubating at pH 7.4 (physiological pH) and 5.0 (lysosomal pH), respectively, and the released Trt-OH was measured using HPLC (Figure 2C).

After 24 h of incubation at pH 7.4, only a small fraction (less than 2%) of *N*-Trt groups was detected in all PRXs. In contrast, complete cleavage of the *N*-Trt groups was observed after 24 h of incubation at pH 5.0. The degradation curves for the peptide-modified and unmodified Me-PRXs largely overlapped under both experimental conditions. The first-order rate constants for Me-PRXs at pH 5.0 and 7.4 were determined according to the previous reports [35]. As a result, the first-order rate constants at pH 5.0 and 7.4 were the similar values among Me-PRX, cRGD-Me-PRX, and cRGE-Me-PRX (Supporting Information Table S1), indicating that the modification of the charged cRGD or cRGE peptides did not affect the stability and dissociation properties of Me-PRXs. Consequently, all Me-PRXs were stable under physiological pH conditions but readily dissociated under acidic conditions.

Another important aspect of Me-PRXs is the ability of released β-CDs to form an inclusion complex with intracellular cholesterol, because the interaction with intracellular cholesterol contributes to the induction of cell death [37]. To assess this, the solubility of cholesterol was measured by incubating with Me-PRXs at pH 7.4 (physiological pH) and 5.0 (lysosomal pH) for 24 h was measured (Figure 2D). At pH 7.4, solubilization of cholesterol was observed when incubated with Me-β-CD and cRGD-Me-β-CD. In contrast, Me-PRXs did not solubilize cholesterol at pH 7.4, because the PRX structure inhibited its interaction with cholesterol. Cholesterol solubility in cRGD-Me-β-CD was lower than that in Me-β-CD. This result indicates that modification with cRGD peptides slightly reduced the affinity for cholesterol, most likely due to steric hindrance. Additionally, the number of methyl groups of cRGD-Me-β-CD (12.5) was less than that of Me-β-CD (14.0), which may explain the reduced cholesterol solubilization capacity. At pH 5.0, the solubilities of cholesterol in the presence of Me-β-CD and cRGD-Me-β-CD were similar to those observed at pH 7.4. However, the solubility of cholesterol in the presence of Me-PRX increased significantly and approached that of the corresponding CD group at pH 5.0. This result indicates that the threaded Me-β-CDs in Me-PRXs were released at pH 5.0 and subsequently formed an inclusion complex with cholesterol. Similar to the Me-β-CD and cRGD-Me-β-CD, the solubility of cholesterol for cRGD-Me-PRX and cRGE-Me-PRX were lower than that for Me-PRX at pH 5.0. Accordingly, the released cRGD-modified Me-β-CDs from cRGD-Me-PRX could solubilize cholesterol by forming an inclusion complex, although the complexation ability was slightly decreased compared to unmodified Me-PRX.

### 3.2. Interaction of cRGD-Me-PRX with Integrin α_v_β_3_

**Figure 3:**
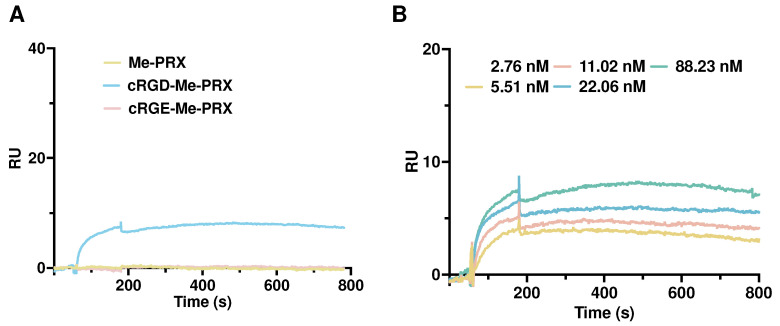
(**A**) SPR sensorgrams for Me-PRX, cRGD-Μe-PRX, and cRGE-Me-PRX on the mouse integrin αvβ3-immobilized sensor chip surfaces at a concentration of 88.23 nM (corresponding to β-CD in PRX). (**B**) SPR sensorgrams for cRGD-Me-PRX at a concentration ranging from 2.76 to 88.23 nM.

To evaluate the affinity of cRGD-Me-PRXs with integrin α_v_β_3_, SPR measurements were performed using recombinant mouse integrin α_v_β_3_ protein-immobilized sensor chips. The SPR sensorgrams of Me-PRX, cRGD-Me-PRX, and cRGE-Me-PRX at the concentration of 88.23 nM (β-CD on PRX) are shown in Figure 3A. The response unit (RU) values increased for cRGD-Me-PRX, whereas negligible changes in RU was observed for cRGE-Me-PRX and Me-PRX. These results indicate that unmodified Me-PRX cannot interact with integrin α_v_β_3_, the non-targeted cRGE moieties cannot recognize integrin α_v_β_3_, and cRGD-Me-PRX can specifically recognize and strongly interact with integrin α_v_β_3_. Next, the effect of cRGD-Me-PRX concentration was evaluated. As a result, the concentration-dependent association of cRGD-Me-PRX with recombinant mouse integrin α_v_β_3_ was observed (Figure 3B). However, the complete dissociation of cRGD-Me-PRX was not observed during the measurements. Therefore, it was difficult to determine the binding parameters. The cRGD peptides in cRGD-Me-PRXs can slide and rotate with CDs on the axial polymers, which increases the mobility and flexibility of the ligands on the PRX backbone, resulting in strong binding to receptor proteins.

### 3.3. Cellular Association and Cytotoxicity of cRGD-Me-PRX

**Figure 4:**
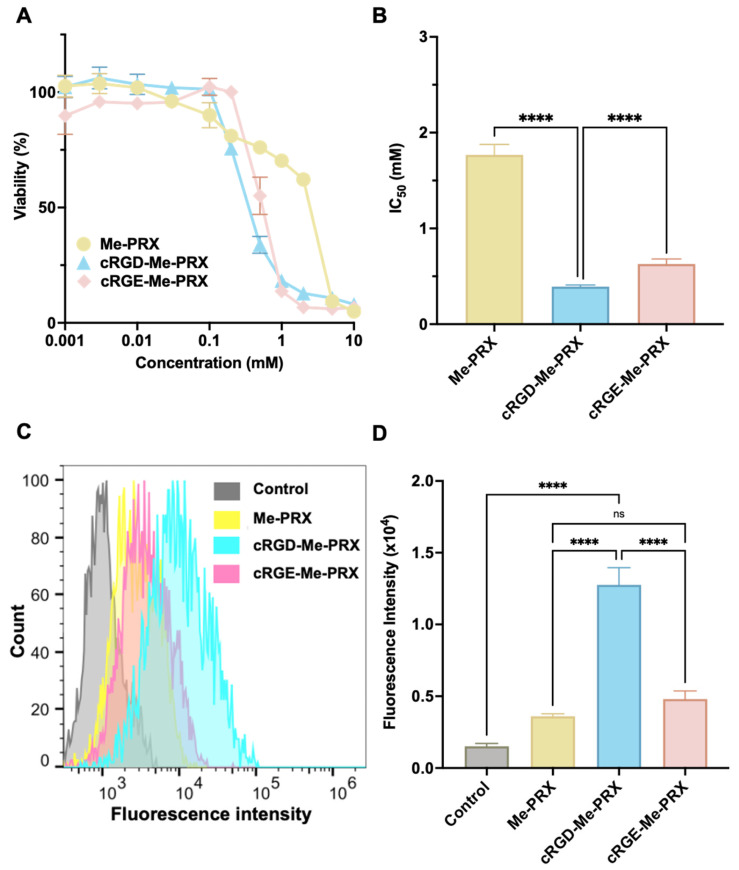
(**A**) Viability curves and (**B**) IC_50_ values of 4T1 cells treated with Me-PRX, cRGD-Me-PRX, and cRGE-Me-PRX for 48 h. The data are expressed as the mean ± SD (n = 4; **** *p* < 0.0001). (**C**) Fluorescence intensity histograms and (**D**) mean fluorescence intensities of 4T1 cells treated with Cy5.5-labeled Me-PRX, cRGD-Me-PRX, and cRGE-Me-PRX (50 μM β-CD) for 3 h at 37 °C. The data are expressed as the mean ± SD (n = 4; **** *p* < 0.0001; ns: not significant).

To evaluate the effect of cRGD modification on the biological functions of Me-PRXs, the cytotoxicity and cellular internalization rate of cRGD-Me-PRX in tumor cells expressing integrin α_v_β_3_. In this study, the evaluations were performed using 4T1 cells, which overexpress integrin α_v_β_3_ (Supporting Information Figure S5). We have previously demonstrated that the cytotoxicity of Me-PRXs is attributed to the intracellular release of threaded Me-β-CD, which stimulates ER stress and autophagic cell death [37]. Cytotoxicity was evaluated by treating 4T1 cells with various concentrations of cRGD-Me-PRXs for 48 h (Figure 4A). Consistent with the results of a previous study [37], the viability of 4T1 cells decreased in a concentration-dependent manner following treatment with Me-PRXs. Among the tested samples, cRGD-Me-PRX induced cell death at lower concentrations than Me-PRX or cRGE-Me-PRX.

For quantitative comparison, half-maximal inhibitory concentration (IC_50_) values were determined from the cell viability curves (Figure 4B). The results revealed that the IC_50_ values of Me-PRXs were considerably lower than those of cRGD-Me-PRX and cRGE-Me-PRX, indicating that peptide modification increased the cytotoxicity of Me-PRXs. The charged functional groups introduced into PRXs can alter their electrostatic interactions with lipids or proteins on cell membranes, thus increasing their uptake by specific cells [50,51]. Thus, the increased cytotoxicity of cRGE-Me-PRX and cRGD-Me-PRX may be due to the presence of charged functional groups in the peptide moieties (Arg, Asp, and Glu), which interact with the cell membrane surface and increase their uptake in 4T1 cells. In addition, the IC_50_ of cRGD-Me-PRX was notably lower than that of cRGE-Me-PRX. The aforementioned SPR results demonstrated that cRGD-Me-PRX can interact with integrin α_v_β_3_, whereas cRGE-Me-PRX cannot. These results suggest that the increased cytotoxicity of cRGD-Me-PRX relies not only on the charged functional group but also on integrin α_v_β_3_-mediated uptake.

Next, the cellular internalization of cRGD-Me-PRXs was assessed to clarify the effect of cRGD modification on cellular internalization. Cellular internalization of cRGD-Me-PRXs was evaluated by flow cytometry using fluorescently labeled cRGD-Me-PRXs (Cy5.5-labeled cRGD-Me-PRXs; Supporting Information Table S1). Note that the number of Cy5.5 molecules modified on Me-PRXs were adjusted to 0.05 to 0.1 per PRX molecule to avoid any effects on the physicochemical properties and cellular uptake of the Me-PRXs. Given the cytotoxicity of Me-PRXs, experiments were performed at a concentration of 100 μM threaded β-CD. 4T1 cells were treated with Cy5.5-labeled Me-PRXs for 3 h, and the fluorescence intensity of the treated cells was measured by flow cytometry. The fluorescence intensities of cRGD-Me-PRX-treated cells were significantly higher than those of cRGE-Me-PRX- and Me-PRX-treated cells (Figure 4C,D). These results indicated that the modification of cRGD increased the cellular uptake efficiency of Me-PRX into 4T1 cells.

**Figure 5:**
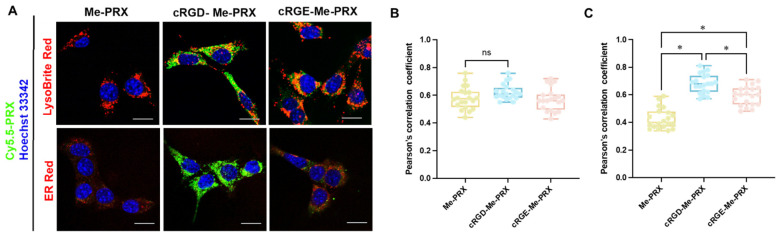
(**A**) CLSM images of 4T1 cells treated with Cy5.5-labeled Me-PRX, cRGD-Me-PRX, and cRGE-Me-PRX (green; 50 μM β-CD) for 3 h at 37 °C (Scale bars: 20 μm). The cell nuclei (blue), endosomes/lysosomes (red), and ER (red) were stained with Hoechst 33342, LysoBrite Red, and ER Red, respectively. (**B**,**C**) Pearson’s correlation coefficients between Cy5.5-labeled PRXs with (**B**) LysoBrite Red or (**C**) ER Red. The data are expressed as a box plot, wherein the upper and lower boundaries of the box indicate the 75th and 25th percentiles, respectively; the lines within the box indicate the median, whereas the whiskers indicate the minimum and maximum (n = 20 cells; * *p* < 0.05; ns: not significant).

To confirm the intracellular distribution of cRGD-Me-PRXs, CLSM was performed 3 h after treatment (Figure 5A). These results confirm that the intracellular uptake of cRGD-Me-PRX was significantly higher than that of Me-PRX and cRGE-Me-PRX. The detailed cellular localization of cRGD-Me-PRXs in 4T1 cells was investigated by staining the endosomes/lysosomes and ER with LysoBrite Red and ER Red, respectively (Figure 5A). The green fluorescence signals derived from Cy5.5-labeled Me-PRX, cRGD-Me-PRX, and cRGE-Me-PRX colocalized with the signals from Lyso Brite Red and ER Red, indicating that all Me-PRXs were localized to endosomes/lysosomes and the ER.

To quantitatively assess the correlation between PRXs and organelles, the Pearson’s correlation coefficient was calculated (Figure 5B,C) [46]. Pearson’s correlation coefficients for all Me-PRXs, LysoBrite, and ER Red were strongly positive, suggesting that Me-PRXs predominantly accumulated in the lysosomes and ER. This observation is consistent with previous findings that Me-PRXs can release threaded Me-β-CDs into lysosomes and preferentially accumulate Me-β-CDs in the ER, leading to the induction of ER stress-mediated autophagic cell death [37]. Among the tested Me-PRXs, Pearson’s correlation coefficients for cRGD-Me-PRXs with the ER were significantly higher. Since the localization of Me-PRX in the ER is critical for inducing autophagic cell death, the high cytotoxicity of cRGD-Me-PRX was attributed to its efficient cellular internalization and subsequent accumulation in the ER.

### 3.4. Involvement of Integrin α_v_β_3_ in the Cellular Internalization of cRGD-Me-PRX

**Figure 6:**
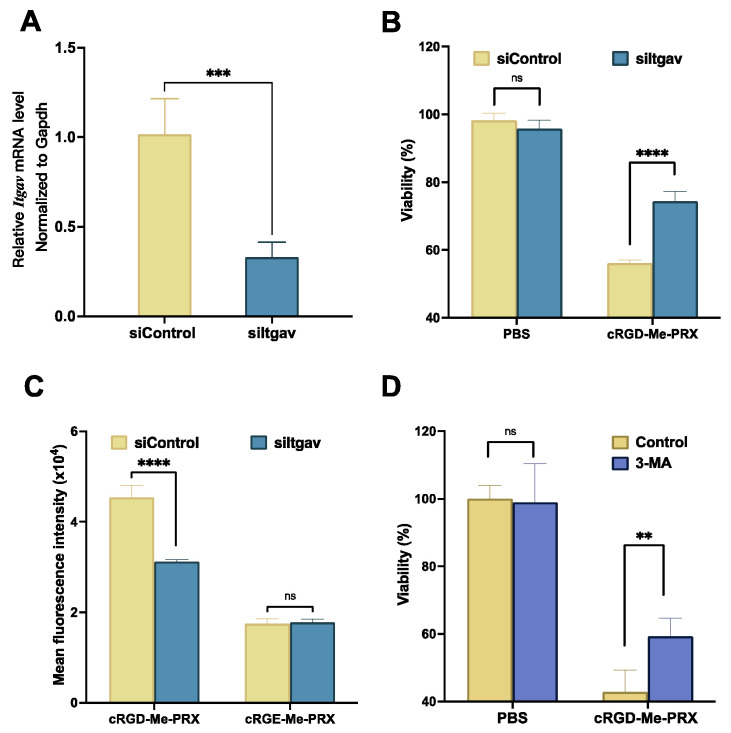
(**A**) The siRNA against the integrin V (siItgav) silencing effect in 4T1 cells after incubation for 24 h before RT-PCR analysis (n = 4; *** *p* < 0.001). (**B**) Fluorescence intensity of α_v_ integrin-downregulated 4T1 cells treated with Cy5.5-cRGE-Me-PRX and Cy5.5-cRGD-Me-PRX at 50 μM threaded β-CD (n = 4; **** *p* < 0.0001; ns: not significant). (**C**) Viability of α_v_ integrin-downregulated 4T1 cells treated with cRGD-Me-PRX at 100 μM threaded β-CD (n = 4; **** *p* < 0.0001; ns: not significant). (**D**) Viability of 4T1 cells treated with cRGD-Me-PRX (100 μM threaded β-CD) in the presence of 3-MA (2 mM) for 48 h (n = 6; ** *p* < 0.01; ns: not significant).

To further confirm the involvement of integrin α_v_β_3_ in the cellular internalization of cRGD-Me-PRX, the expression level of integrin α_v_ was downregulated using siRNA (Figure 6A). By the treatment of 4T1 cells with siRNA against integrin α_v_ (siItgav), the expression level of integrin α_v_ was decreased to 32.9% compared with that 4T1 cells treated with non-targeting siRNA (siControl). When 4T1 cells were pretreated with siItgav, the viability of 4T1 cells treated with cRGD-Me-PRX was significantly higher than that of 4T1 cells pretreated with siControl (Figure 6B). Flow cytometric analysis showed that pretreatment with siItgav decreased the cellular uptake of cRGD-Me-PRX, whereas the fluorescence intensity of non-targeting cRGE-Me-PRX was not affected (Figure 6C). These results strongly suggest that the binding of cRGD peptides to integrins α_v_β_3_ plays a crucial role in the cellular internalization of cRGD-Me-PRX. However, the cellular uptake of cRGD-Me-PRX was not completely inhibited by siItgav. We have previously studied the cellular uptake pathway of Me-PRX and other PRX derivatives [35,37,38]. Me-PRX and other PRX derivatives are essentially internalized into cells via various endocytosis pathways including clathrin-mediated endocytosis, caveolae-mediated endocytosis, and micropinocytosis. Therefore, we considered that cRGD-Me-PRX internalized into cells via Integrin-mediated endocytosis and other non-specific endocytosis pathways.

Previous studies have demonstrated that Me-PRX induces autophagic cell death [37,38]; therefore, it is important to determine whether cRGD-Me-PRX-treated 4T1 cells undergo autophagic cell death. 3-Methyladenine (3-MA) is a commonly used inhibitor of autophagy that functions by inhibiting class III phosphoinositide 3-kinase (PI3K) activity, which plays a pivotal role in autophagy induction [52]. Previous studies have shown that inhibition of autophagy using 3-MA increases the viability of Me-PRX-treated cells [37,38]. To exclude the effects of 3-MA on 4T1 cell viability, cRGD-Me-PRX-treated 4T1 cells were tested for the induction of autophagic cell death at a lower concentration of 3-MA (2 mM), which did not affect cell viability (Figure 6D). 3-MA restored the viability of cRGD-Me-PRX treated 4T1 cells, confirming that cRGD-Me-PRX induced autophagic cell death, similar to that induced by Me-PRX.

### 3.5. Biodistribution and Tumor Accumulation of cRGD-Me-PRX

**Figure 7:**
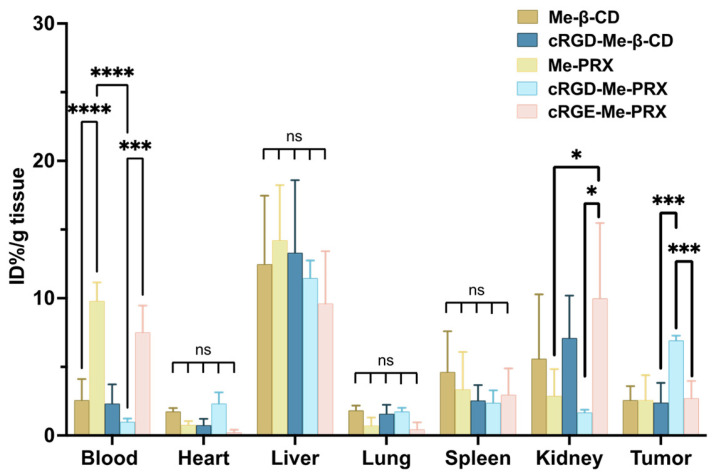
Body distribution of Cy5.5-labeled Me-β-CD, cRGD-β-CD Me-PRX, cRGE-Me-PRX, and cRGD-Me-PRX in 4T1 tumor-bearing mice after 24 h of i.v. administration at 500 mg/kg (n = 6, * *p* < 0.05, *** *p* < 0.001, and **** *p* < 0.0001; ns: not significant).

To investigate the selective accumulation of cRGD-Me-PRX in tumor tissues, its biodistribution after intravenous (i.v.) administration in mice bearing 4T1 tumor was examined. After 24 h after i.v. administration of Cy5.5-labeled samples, the percentage of the initial dose (ID%) in the blood, major organs (heart, liver, lungs, spleen, and kidneys), and tumors was determined by analyzing the fluorescence intensities of the tissue extracts (Figure 7). The results revealed that Me-β-CD and cRGD-Me-β-CD mostly accumulated in the kidney, indicating that these samples were excreted from the kidney owing to their low molecular weight. However, macromolecular Me-PRX remained in the blood, likely because of the significantly improved blood circulation time owing to its large molecular weight. Interestingly, increased accumulation in the tumor and decreased blood concentration of cRGD-Me-PRXs were observed. The level of cRGE-Me-PRX accumulation in the tumors was comparable to that of Me-PRX. These findings suggested that the introduction of the cRGD peptide was recognized by tumor cells, which contributed to enhancing the tumor accumulation of cRGD-Me-PRX. Although cRGD-Me-β-CD also have cRGD-peptide, its accumulation level in tumor tissue was low, most likely due to its renal clearance [53].

### 3.6. Antitumor Effects of cRGD-Me-PRX in the 4T1 Tumor-Bearing Mice Model

**Figure 8:**
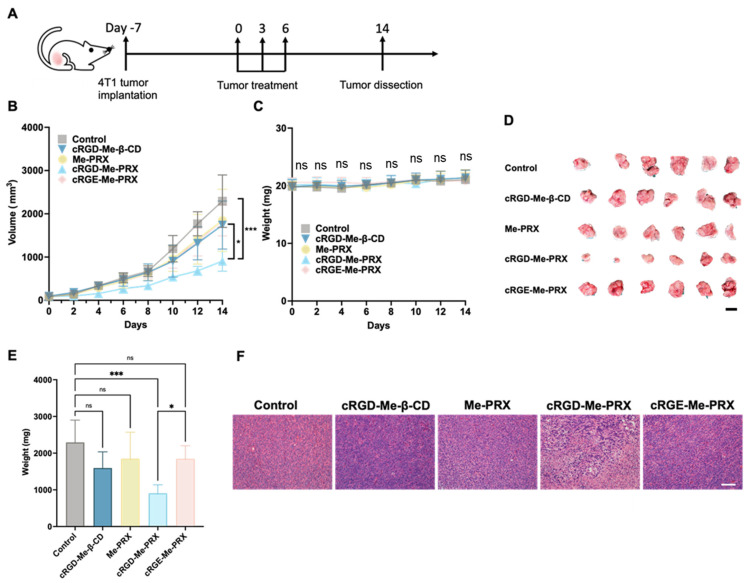
(**A**) Schematic illustration on the treatment of subcutaneous 4T1 tumor-bearing mice. (**B**) Average tumor growth and (**C**) body weight of mice treated with cRGD-Me-β-CD, Me-PRX, cRGD-Me-PRX, and cRGE-Me-PRX. The data are expressed as the mean ± SD (n = 6; ns: not significant among all groups). (**D**) Representative photograph of dissected tumor tissues (scale bar: 2 cm), and (**E**) average weight or tumors collected from mice treated with cRGD-β-CD, Me-PRX, cRGD-Me-PRX, and cRGE-Me-PRX. (**F**) H&E staining of 4T1 tumor sections at 14 days after the treatment (scale bar: 100 μm). The data are expressed as the mean ± SD (n = 6; * *p* < 0.05; *** *p* < 0.001; ns: not significant).

Due to the enhanced cellular internalization, cytotoxicity, and tumor accumulation of cRGD-Me-PRX, it is thought to have an excellent antitumor effect. The 4T1 tumor model is commonly used to investigate the efficacy of integrin-binding antitumor drugs because these tumors are highly angiogenic and overexpress integrin receptors [54,55,56]. To demonstrate the in vivo antitumor effects of cRGD-Me-PRX, BALB/c mice bearing 4T1 tumor was utilized in this study. These mice were intravenously injected three times with saline (control), cRGD-Me-β-CD, Me-PRX, cRGD-Me-PRX, or cRGE-Me-PRX at 30 mg/kg, and tumor size was measured (Figure 8A). As shown in Figure 8B, the control group (saline) showed rapid tumor growth. Administration of cRGD-Me-β-CD, Me-PRX, or cRGE-Me-PRX did not significantly suppress tumor growth. However, tumor growth was significantly retarded in the cRGD-Me-PRX-treated group (Figure 8B, Supporting Information Figure S6) [57,58,59,60]. Mice treated with cRGD-Me-PRX had the lightest tumor weight after 14 d of feeding (Figure 8C). Additionally, the administration of cRGD-Me-PRX did not affect the body weight of the mice for 14 d (Figure 8D,E). Tumor weight measurements at 14 d reveled that the cRGD-Me-PRX group had significantly lower tumor weights than the control group. These results suggested that cRGD-Me-PRX has potent antitumor activity without marked side effects. We considered that the antitumor effect of cRGD-Me-PRX could be attributed to its enhanced accumulation at the tumor site (Figure 7).

To further evaluate the antitumor efficacy of cRGD-Me-PRX, tumor tissues were dissected and H&E staining of tissue sections was performed (Figure 8F). H&E images of the control group showed a large number of tumor cells with relatively regular cellular morphology. Similar images were obtained for the cRGD-Me-β-CD, Me-PRX, and cRGE-Me-PRX groups. In contrast, the cRGD-Me-PRX group showed a significant decrease in the number of tumor cells in the microscopic field, with fragmented or absent nuclei, extensive tissue necrosis, and nuclear shrinkage and fragmentation. These results confirm that cRGD-Me-PRX effectively induces cell death, leading to the suppression of tumor growth.

### 3.7. Biosafety Evaluation of Me-PRXs and Me-β-CDs

**Figure 9:**
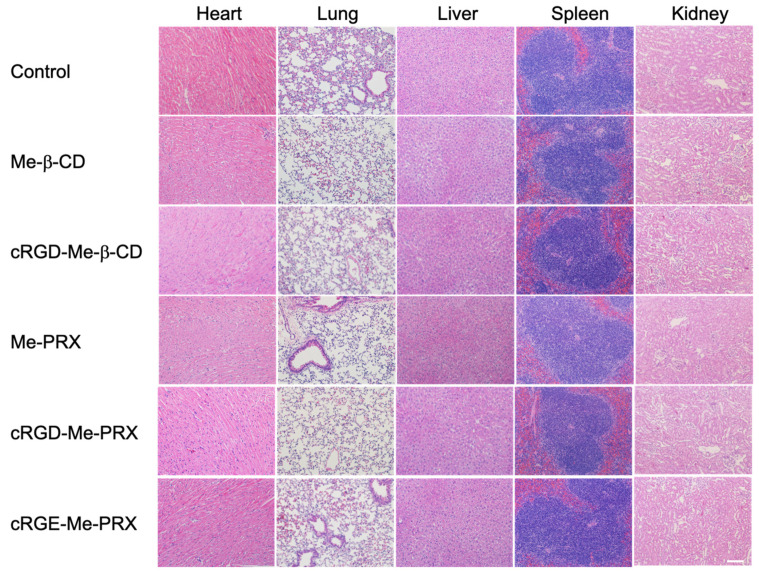
Histologic assessments of main organs with H&E staining in normal mice after 24 h of i.v. administration of Me-β-CD, Me-PRX, cRGD-Me-β-CD, cRGD-Me-PRX, and cRGE-Me-PRX at 500 mg/kg (scale bar: 100 μm).

To further ensure the biocompatibility of cRGD-Me-PRXs, the samples were administered intravenously at a dose of 500 mg/kg to normal mice. The mice injected with Me-PRXs and Me-β-CDs did not die. Histological examination of the fixed tissues showed no significant morphological changes in the isolated organs in the control group compared with those in mice treated with Me-PRXs and Me-β-CDs (Figure 9). These results confirm that Me-PRXs and Me-β-CDs are sufficiently biocompatible in vivo.

**Figure 10:**
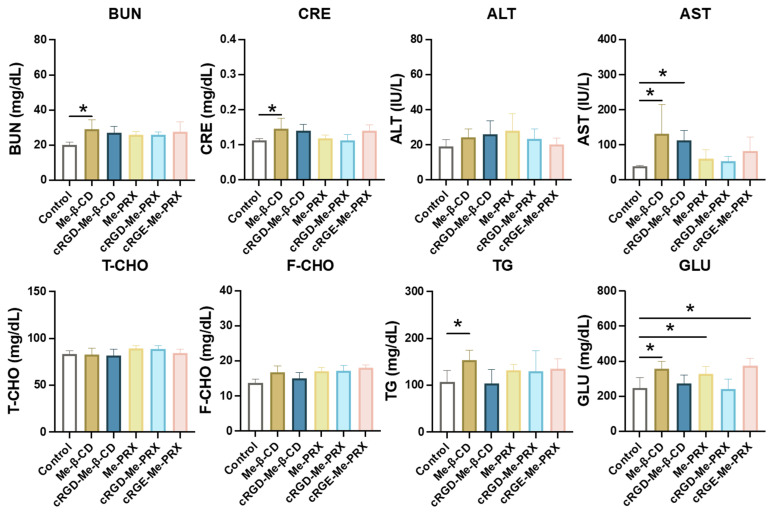
Blood biochemical analysis of BALB/c mice after administration of saline, Me-β-CD, cRGD-Μe-β-CD, Me-PRX, cRGD-Me-PRX, and cRGE-Me-PRX at a dose of 500 mg/kg. BUN: blood urea nitrogen; CRE: creatinine; ALT: aspartate aminotransferase; AST: alanine transaminase; F-CHO: free cholesterol; E-CHO: esterified cholesterol; TG: triglycerides; GLU: glucose. Data are expressed as the mean ± SD (n = 6; * *p* < 0.05).

Next, hepatic toxicity, renal toxicity, and blood metabolic abnormalities were assessed in mice administered with Me-PRXs and Me-β-CDs because Me-β-CD can induce hepatic toxicity, renal toxicity, and erythrocyte hemolysis [30,61]. Plasma concentrations of renal function parameters (BUN and CRE), liver enzymes (AST and ALT), and metabolic parameters (F-CHO, E-CHO, TG, and GLU) were measured 24 h after administration (Figure 10). Note that the administration of Me-β-CD increased the concentration of BUN, CRE, and AST significantly, indicating its hepatic and renal toxicities. In contrast, the plasma concentrations of renal function parameters, liver enzymes, and metabolic parameters remained unchanged after administration of Me-PRX, cRGD-Me-PRX, and cRGE-Me-PRX. These results suggest that the toxicity of Me-β-CD in normal organs were mitigated by PRX structure. Consequently, we concluded that cRGD-Me-PRX is a promising candidate for the cancer chemotherapy using Me-β-CD due to its excellent antitumor effect and sufficient biosafety.

## 4. Conclusions

In this study, cRGD-Me-PRX was prepared by orthogonal post-modification of the cRGDfk peptide via click chemistry for the targeted delivery of Me-PRX in tumor tissues. cRGD-Me-PRX showed higher cytotoxicity and cellular internalization in 4T1 cells than Me-PRX and cRGE-Me-PRX, due to its ability to recognize integrin α_v_β_3_. Additionally, cRGD-Me-PRXs showed high tumor accumulation in a mouse model, leading to antitumor effects. Furthermore, cRGD-Me-PRX did not adversely affect normal organs, suggesting excellent biocompatibility and safety in vivo. The use of cRGD conjugation to achieve selectivity enhancement to integrin α_v_β_3_-positive cancer cells is a promising design strategy for Me-PRX toward antitumor therapy. The unique autophagic cell death mechanism of Me-PRXs provides an opportunity for optimal design and combination with other chemotherapeutic drugs based on different cell death mechanisms to achieve even greater antitumor effects. These findings have significant implications for the development of targeted cancer therapies and highlight the potential of cRGD-Me-PRXs for future clinical applications.

## Figures and Tables

**Table 1:** Characterization of β-CDs and PRXs.

Code ^a^	Precursor PRXs ^b^	Number of Threaded β-CD	Number of Methyl Groups per PRX ^c^	Number of Peptides per PRX ^c^	Conversion Ratio of Ligand	*M*_n_ ^d^
Me-β-CD	–	–	14.0	–	–	1310
cRGD-Me-β-CD	–	–	12.4	0.75	–	1840
Me-PRX	PRX	10.5	147.0 (14.0)	–	–	21,500
cRGE-Me-PRX	N_3_-PRX	14.9	184.9 (12.4)	11.1	76.6%	34,600
cRGD-Me-PRX	N_3_-PRX	14.9	184.9 (12.4)	11.3	77.9%	34,600

^a^ *X*-Me-PRX, where X denotes the type of peptide-modified Me-PRX. ^b^ Y-N_3_-PRX, where Y denotes the number of azido groups on PRX calculated from ^1^H NMR spectra in DMSO-*d_6_*. ^c^ calculated from ^1^H NMR spectra in D_2_O.The values in parentheses denote the number of modified Me groups per threaded β-CD molecule. ^d^ Calculated based on the chemical composition of Me-PRXs, as determined by ^1^H NMR spectroscopy.

## Data Availability

The data presented in this study are available on request from the corresponding author.

## Supplementary Materials

The following supporting information can be downloaded at: https://www.mdpi.com/article/10.3390/biom14020223/s1, Figure S1: ^1^H NMR spectrum of Fmoc-Lys(1-pentynoyl)-OH; Figure S2: HPLC charts and ESI-MS spectra of butynyl-cRGDfK and butynyl-cRGEfK; Figure S3: SEC charts of Me-PRX, N_3_-PRX, and Me-N_3_-PRX; Figure S4: ^1^H NMR and FT-IR spectra of Me-PRX and cRGE-Me-PRX; Figure S5: flow cytometry analysis of 4T1 cells; Figure S6: individual tumor volumes of 4T1 tumor-bearing mice; Table S1: First-order rate constants for the cleavage of the *N*-Trt groups of Me-PRXs; Table S2: characterization of Cy5.5-labeled Me-PRXs.
