# Supplementary material for: Enhanced Tumor Targeting and Antitumor Activity of Methylated β-Cyclodextrin-Threaded Polyrotaxanes by Conjugating Cyclic RGD Peptides
Source: Biomolecules. 2024 Feb 15;14(2):223. doi: 10.3390/biom14020223 (PMC10886891; doi:10.3390/biom14020223)
Supplement: Supplementary file 1 [file biomolecules-14-00223-s001.zip › biomolecules-2856301-supplementary.pdf]

Supplementary Materials

Enhanced Tumor Targeting and Antitumor Activity of Methylated β-Cyclodextrin-Threaded Polyrotaxanes by Conjugating Cyclic RGD Peptides

Shunyao Zhang, Atsushi Tamura * and Nobuhiko Yui

Department of Organic Biomaterials, Institute of Biomaterials and Bioengineering, Tokyo Medical and Dental University (TMDU), 2-3-10 Kanda-Surugadai, Chiyoda, Tokyo 101-0062, Japan

* Correspondence: tamura.org@tmd.ac.jp

Table of contents

S1. Materials
S2. Instrumentation
S3. Synthesis and characterization of butynyl-cRGDfK
S4. Fluorescence labelling of PRXs
S5. References

S1. Materials

Benzotriazol-1-yloxytripyrrolidinophosphonium hexafluorophosphate (PyBOP), 1-[bis(dimethylamino)methylene]-1H-1,2,3-triazolo[4,5-b]pyridinium 3-oxide hexafluorophosphate (HATU), *N,N*-diisopropylethylamine (DIPEA), *N*-(3-Dimethylaminopropyl)-*N'*-ethylcarbodiimide hydrochloride (EDC), hydroxybenzotriazole (HOBt), *N*-hydroxysuccinimide (NHS), and 4-pentynoic acid were obtained from Tokyo Chemical Industry (Tokyo, Japan). 2-Chlorotrityl chloride resin (2-Cl-Trt resin) and reagents for peptide synthesis were obtained from Watanabe Chemical (Osaka, Japan). All other reagents and solvents were obtained from Fujifilm Wako Pure Chemical and Kanto Chemical (Tokyo, Japan).

S2. Instrumentation

^{1}H nuclear magnetic resonance (NMR) spectra were recorded using a Bruker Avance III 400 MHz spectrometer (Bruker BioSpin, Rheinstetten, Germany) in DMSO-d_6 at 25 °C. Chemical shifts in ^{1}H NMR spectra were referenced using DMSO (2.5 ppm in DMSO-d_6) and HDO (4.65 ppm in D_2O). The UV–vis absorption of the solutions was measured using a V-550 UV–vis spectrophotometer (Jasco, Tokyo, Japan). Electrospary ionization mass spectrometry (ESI-MS) was used on a microTOF focus II (Bruker Daltonics, Bremen, Germany).

S3. Synthesis and characterization of butynyl-cRGDfK

Fmoc-Lys(1-pentynoyl)-OH was prepared as followed [S1]. 4-Pentynoic acid (0.303 g, 3.08 mmol), EDC (1.18 g, 6.17 mmol) and NHS (0.71 g, 6.17 mmol) were dissolved in 30 mL THF/CH_2Cl_2 (volume ratio =1:2). The mixture was then stirred overnight at room temperature. Subsequently, aqueous $NaHCO_3$ solution was added. The mixture was extracted with ethyl acetate for 3 times, washed with brine, and dried over Na_2SO_4. The desiccant was filtered and concentrated to obtain an oily product. Fmoc-Lys-OH·HCl (2.0 g, 4.94 mmol) was suspended in 25 mL water/1,4-dioxane (volume ratio =1:1), then $NaHCO_3$ (0.415 g, 4.94 mmol) were added to the solution and stirred overnight at room temperature. Upon completion of the reaction, the mixture was concentrated in vacuo and the residue was extracted with ethyl acetate for three times. The mixture was washed with brine and dried over Na_2SO_4. The crude product was purified by column chromatography in ethyl acetate/hexane (volume ratio = 3:2) with 5% acetic acid. This afforded compound as a white solid (1.08g, Yield 78.56%). The ^{1}H NMR spectrum of Fmoc-Lys(1-pentynoyl)-OH in DMSO-d_6 was measured (Figure S1), and ESI-MS was performed to determine the exact mass of Fmoc-Lys(1-pentynoyl)-OH (*m/z* calculated for $C_{26}H_{27}N_2O_5$ [M-H]$^-$): 447.19, found: 447.15).

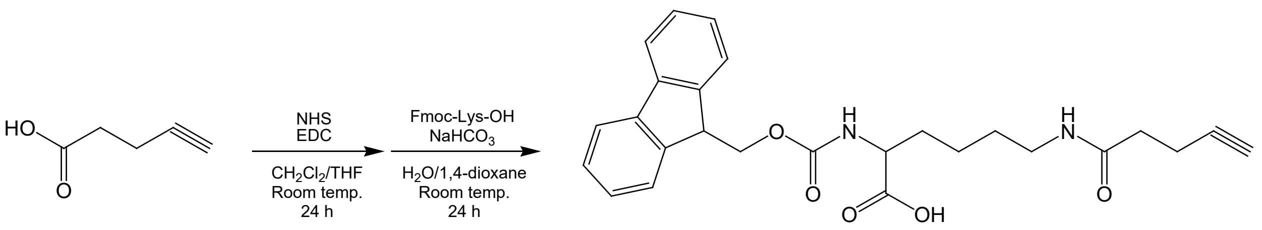

Scheme S1. Scheme for synthesis of Fmoc-Lys(1-pentynoyl)-OH.

The peptide was prepared using the standard Fmoc solid-phase peptide synthesis method using 2-Cl-Trt resin [S2, S3]. The resins were first swollen in CH_2Cl_2 for 1 h and then washed five times with CH_2Cl_2. Linear RGDfk was assembled *via* consecutive reactions with Fmoc-Lys(1-pentynoyl)-OH (1.5 eq. to active chloride on the resin), and DIPEA (4 eq.) in DMF for 1.5 h at room temperature. The resin was washed five times with CH_2Cl_2. A solution of CH_2Cl_2:MeOH:DIPEA (volume ratio = 17:2:1) was the added to mask the unreacted functional groups on the resin. All Fmoc groups were removed with 20% piperidine in DMF and washed thoroughly with DMF. The linear pentapeptide RGDfK was assembled using standard Fmoc procedures by consecutive addition of the protected amino acids (3 eq.), HATU (3 eq.) and DIPEA (6 eq.) in DMF. Fmoc-D-Phe-OH, Fmoc-Asp(OtBu)-OH, Fmoc-Gly-OH, Fmoc-Arg(Pbf)-OH were added sequentially. After adding the last amino acid, the Fmoc group was removed using 20% piperidine in DMF and washed thoroughly with DMF and CH_2Cl_2. The synthesized linear RGDfK was then cut from the resin for 1 h using cleavage cocktail (acetic acid:trifluoroethanol:CH_2Cl_2 = 1:1:3 volume ratio). Finally, the resin was washed with CH_2Cl_2, and the protected linear RGDfK was obtained by precipitation with cold ether.

Liquid-phase cyclization was conducted in a CH_2Cl_2 containing 1 mM linear RGDfK, PyBOP (3eq.), HOBt (3eq.), and DIPEA (3eq.) was then added to the solution and stirred for 16 h at room temperature. After the reaction, the raw product was precipitated and washed with cold water. Another cocktail containing trifluoroacetic acid:water:triisopropylsilane (volume ratio = 95:2.5:2.5) was used for the total deprotection reaction and stirred for 2 h at room temperature. The solution was concentrated and precipitated in cold diethyl ether. The product (butynyl-cRGDfK) was obtained *via* filtration and dried completely. The control butynyl-cRGEfK peptide was synthesized using similar method**,** but Asp(OtBu)-OH was substituted with Glu(OtBu)-OH.

The purity butynyl-cRGDfK and butynyl-cRGEfK of was analyzed using a high-performance liquid chromatography (HPLC) setup consisting of an AS-950 autosampler, PU-4180 pump, CO-965 column oven, UV-970 detector (Jasco, Tokyo, Japan), and a combination of Cosmosil 5C18-AR-II packed column (250 mm × 4.6 mm internal diameter) and Cosmosil 5C18-AR-II guard column (10 mm × 4.6

mm internal diameter; Nacalai Tesque, Kyoto, Japan). The mobile phase consisted of acetonitrile containing 0.1% trifluoroacetic acid (TFA; solvent A) and distilled water containing 0.1% trifluoroacetic acid (solvent B). The samples were eluted using a linear gradient program at a flow rate of 1.0 mL/min at 40 °C and detected at 220 nm. The linear gradient program started from A:B = 10:90 to 60:40 for 30 min. Because the purity of the peptides was sufficiently high (>95%), they were used without further purification (**Fig. S2A, 2B**). ESI-MS was performed to determine the exact masses of butynyl-cRGDfK (**Fig. S2C**; *m/z* calculated for $C_{32}H_{46}N_9O_8$ $[M+H]^+$): 684.35, found: 684.35) and butynyl-cRGEfK (**Fig. S2D**; *m/z* calculated for $C_{33}H_{48}N_9O_8$ $[M+H]^+$): 698.35, found: 698.36)

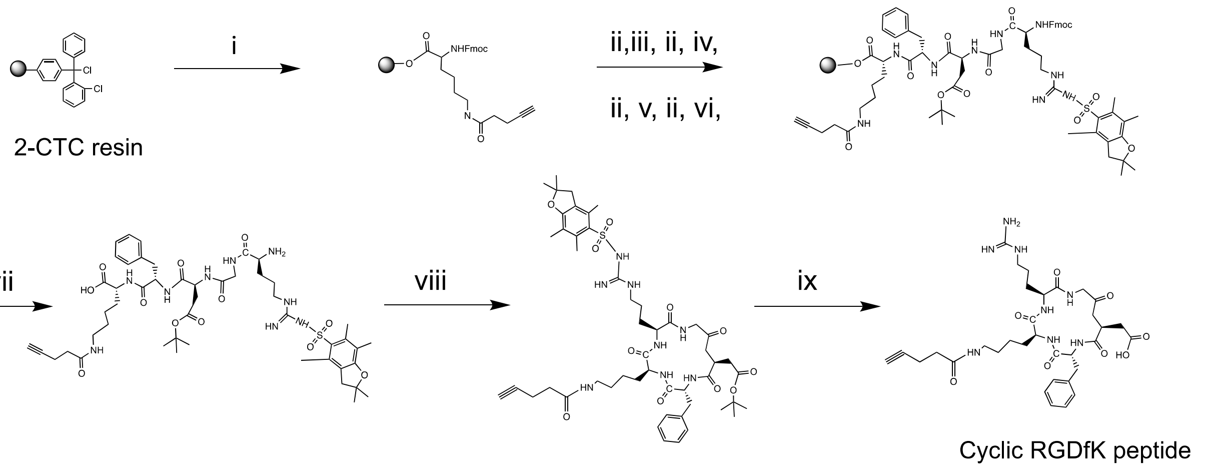

Scheme S2. Schematic for synthesis of cRGDfK. Reagents and conditions:(i) Fmoc-Lys(1-pentynoyl)-OH, DIPEA, CH_2Cl_2, rt, 1.5 h; (ii) piperidine:DMF (1:4), rt, 10 min; (iii) Fmoc-D-Phe-OH, HATU, DIPEA, rt, 1.5 h; (iv) Fmoc-Asp(OtBu)-OH, HATU, DIPEA, rt, 1.5 h; (v) Fmoc-Gly-OH, HATU, DIPEA, rt, 1.5 h; (vi) Fmoc-Arg(Pbf)-OH, HATU, DIPEA, rt, 1.5 h (vii) acetic acid:trifluoroethanol:CH_2Cl_2=1:1:3; rt, 1 h; (viii). PyBOP, HOBt, DIPEA, rt, 16h; (ix) trifluoroacetic acid:water:triisopropylsilane = 95:2.5:2.5; rt, 2 h;

S4. Fluorescence labelling of PRXs

The Cy5.5 labeled cRGD-Me-PRX was prepared as described previously [S4]. The cRGD-Me-PRX (100 mg) and CDI (2 eq. to PRX, 0.95 mg, 5.85 μmol) was dissolved in dehydrated DMF and stirred for 6h at room temperature under N_2. Then, triethylamine (3 eq. to PRX, 1.22 μL, 8.77 μmol) and Cy5.5 amine (1 eq. to PRX, 2.52 mg, 2.92 μmol) were added and the mixture was stirred at room temperature for 24 h. It was then purified by dialysis against methanol for three days (MWCO: 6000–8000) and pure water for two days. Finally, the solution was freeze-dried to yield Cy5.5-modified

cRGD-Me-PRX (Cy5.5-cRGD-Me-PRX, 92 mg, 90.8% yield). Cy5.5-Me-PRX and Cy5.5-cRGE-Me-PRX were prepared using the same method. The UV–vis absorption spectra of Cy5.5-Me-PRXs in DMF were measured using a V-550 UV–vis spectrophotometer. The number of Cy5.5 modified onto the Me-PRXs was calculated from the absorbance at 684 nm using the molar absorption coefficient of Cy5.5 in DMF (ε = 64,400). The characteristics of Cy5.5-cRGD-Me-PRX are summarized in Table S2. Because the number of Cy5.5 varied among the samples (0.024 to 0.067 per PRX), Cy5.5-Me-PRXs were combined with non-labeled Me-PRXs to adjust the fluorescence intensities among the samples for cell and animal experiments.

References

S1 K. Shinoda, Y. Sohma and M. Kanai, *Bioorg. Med. Chem. Lett.*, 2015, **25**, 2976–2979.

S2 X. Dai, Z. Su and J. O. Liu, *Tetrahedron Lett.*, 2000, **41**, 6295-6298.

S3 C. F. McCusker, P. J. Kocienski, F. T. Boyle and A. G. Schätzlein, *Bioorg. Med. Chem. Lett.*, 2002, **12**, 547–549.

S4 M. Ohashi, A. Tamura and N. Yui, *Langmuir*, 2021, **37**, 11102–11114.

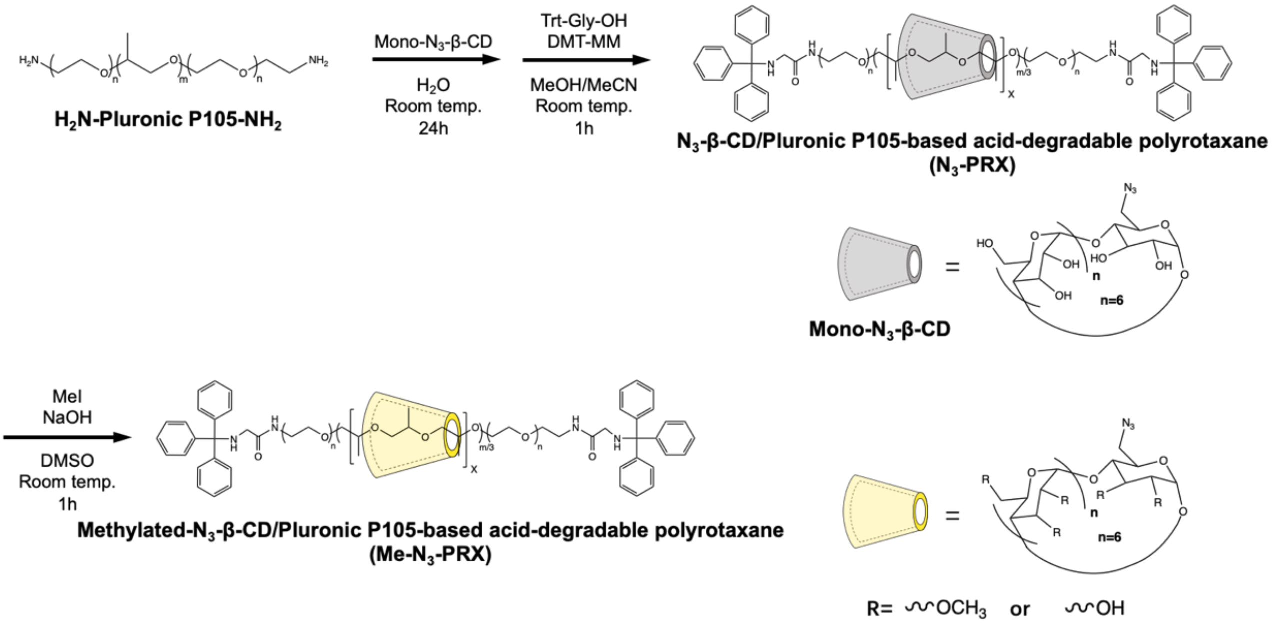

Scheme S3. Scheme for synthesis of acid-degradable methylated monoazido-β-CD/PEG-*b*-PPG-*b*-PEG (Pluronic P105) polyrotaxane (Me-N_3-PRX), where *n*, *m*, *x* denote the number of monomer units in PEG, the number of monomer units in PPG, and the number of threaded β-CD in PRX, respectively.

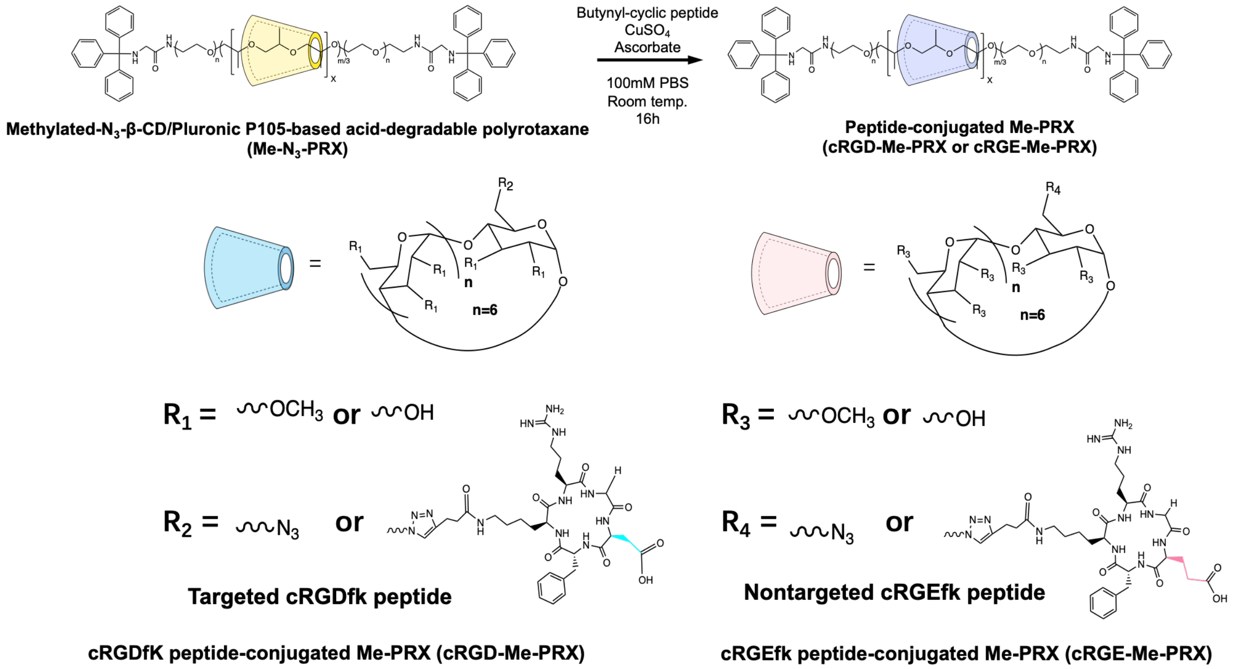

Scheme S4. Scheme for synthesis of cRGD-Me-PRX and cRGE-Me-PRX.

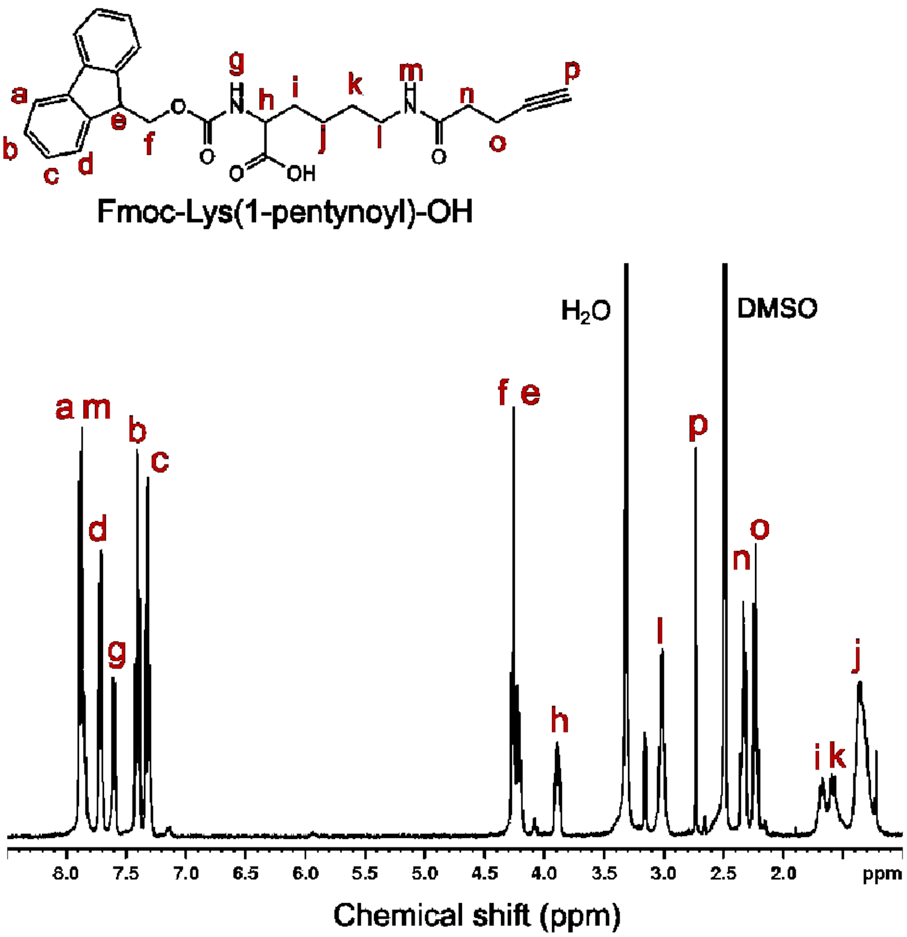

Figure S1. ^1H NMR spectrum of Fmoc-Lys(1-pentynoyl)-OH in DMSO-d_6 at 25 °C.

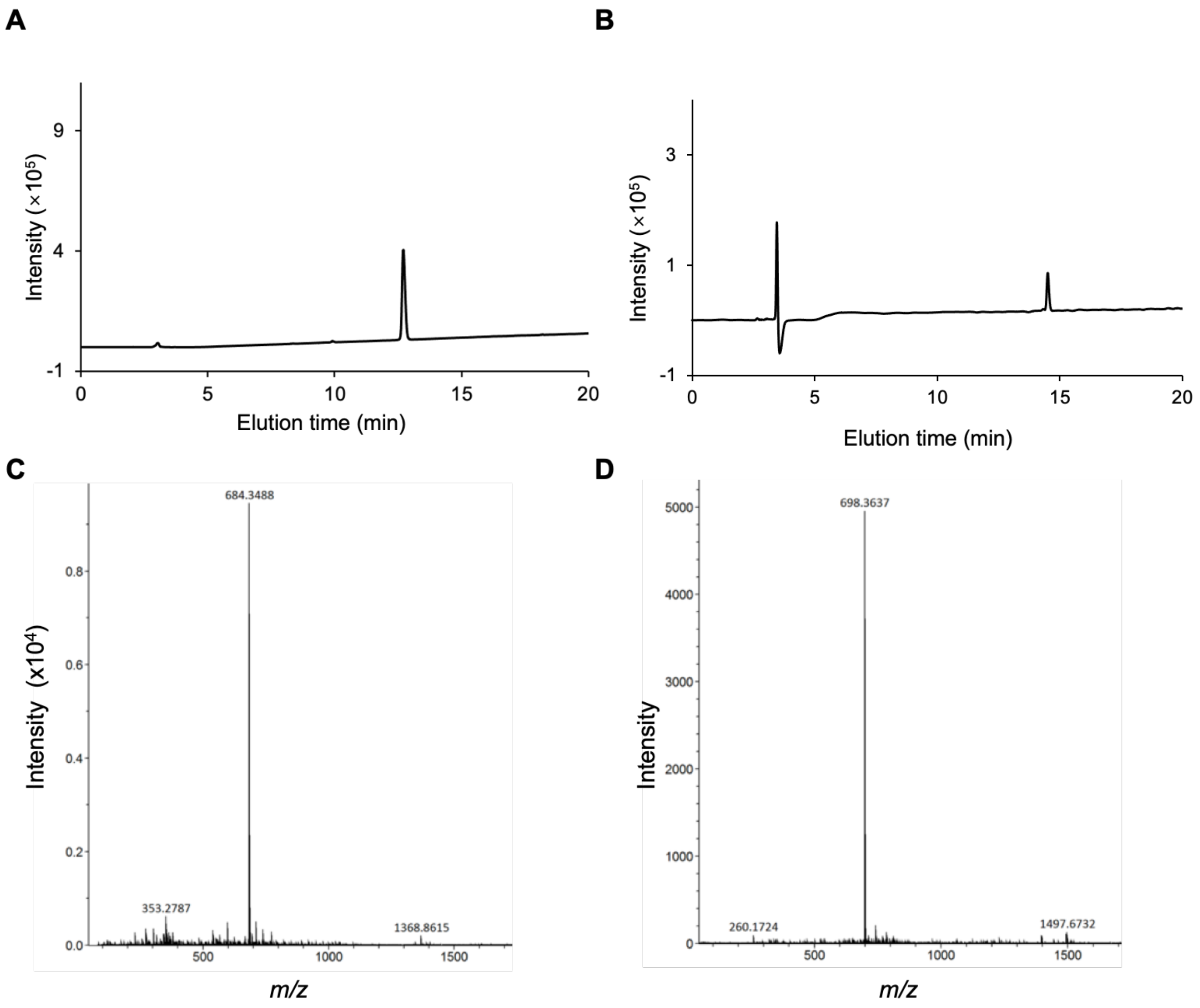

Figure S2. (A, B) HPLC charts of (A) butynyl-cRGDfK and (B) butynyl-cRGEfK. (C, D) ESI-MS spectra of (C) butynyl-cRGDfK and (D) butynyl-cRGEfK.

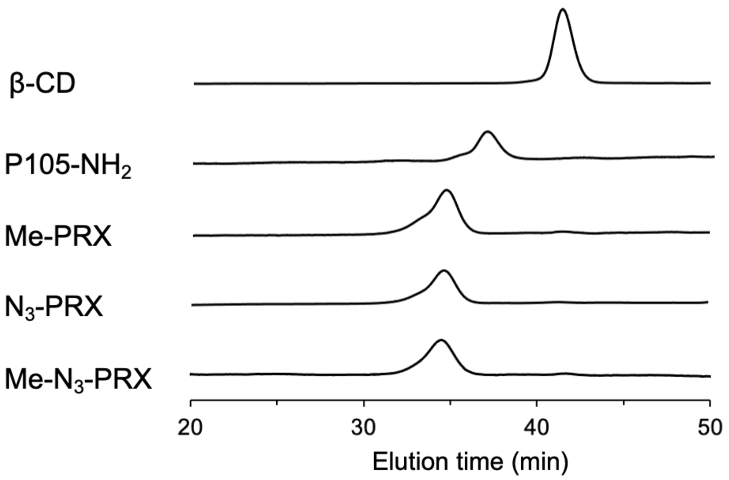

Figure S3. SEC charts of free β-CD, H_2N-PEG-*b*-PPG-*b*-PEG-NH_2 axis polymer (P105-NH_2), Me-PRX, N_3-PRX, and Me-N_3-PRX in DMSO.

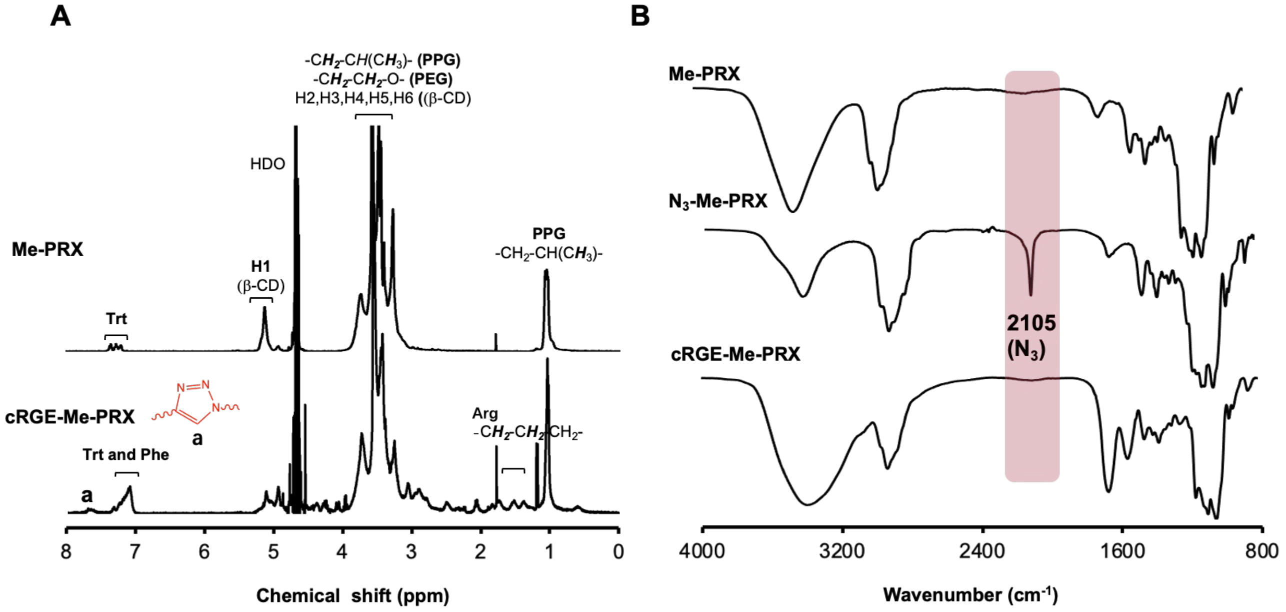

Figure S4. (A) ^{1}H NMR spectra of Me-PRX and cRGE-Me-PRX in D_2O at 25 °C. (B) FT-IR spectra of Me-PRX, N_3-Me-PRX, and cRGE-Me-PRX.

Table S1. First-order rate constants for the cleavage of the *N*-Trt groups of Me-PRXs at 37 ºC.

Sample	k at pH 5.0 (h^{-1})	k at pH 7.4 (h^{-1})
Me-PRX	2.1×10^{-1}	3.2×10^{-3}
cRGD-Me-PRX	2.1×10^{-1}	3.5×10^{-3}
cRGE-Me-PRX	2.6×10^{-1}	2.9×10^{-3}

Table S2. Characterization of Cy5.5-labeled-Me-PRXs.

Sample	Precursor PRXs	Number of Cy5.5 on PRX[a]
Cy5.5-Me-PRX	Me-PRX	0.024
Cy5.5-cRGD-Me-PRX	cRGD-Me-PRX	0.051
Cy5.5-cRGE-Me-PRX	cRGE-Me-PRX	0.067

[a] Determined using UV–vis spectrometry

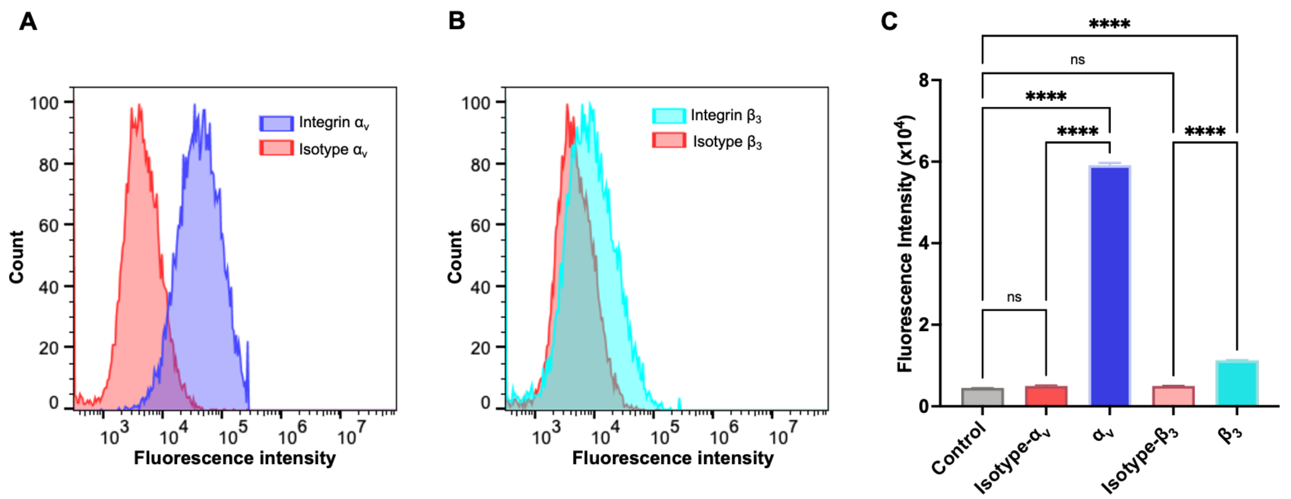

Figure S5. (A, B) Fluorescence intensity histograms of 4T1 cells treated with isotype control and PE-anti-Integrin α_v antibodies and (B) isotype control and PE-anti-Integrinβ_3 antibodies. (C) Mean fluorescence intensities of 4T1 cells treated with isotype α_v control, PE-anti-Integrin α_v-antibody, isotype β_3 control, and PE-anti-Integrin β_3-antibody. (n = 3, ****$P < 0.0001$, ns: not significant).

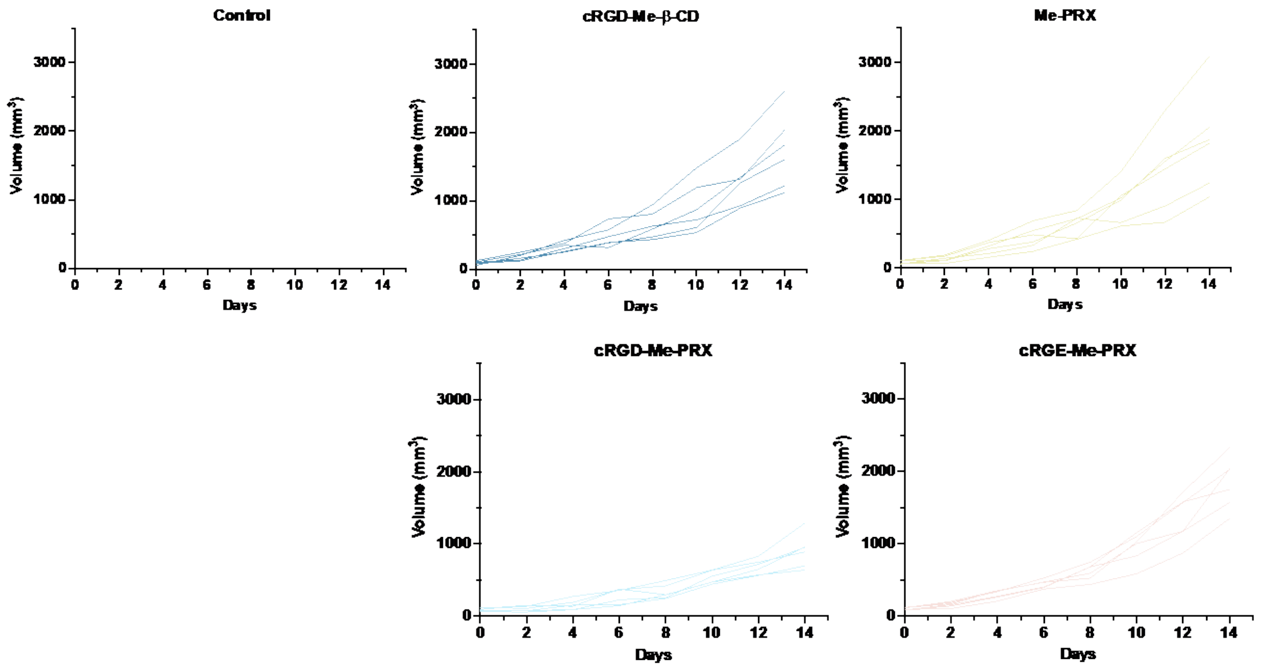

Figure S6. Individual tumor volumes of 4T1 tumor bearing mice treated with saline, cRGD-Me-β-CD, Me-PRX, cRGD-Me-PRX, and cRGE-Me-PRX.
